# Transgenic *Plasmodium *parasites stably expressing *Plasmodium vivax *dihydrofolate reductase-thymidylate synthase as *in vitro *and *in vivo *models for antifolate screening

**DOI:** 10.1186/1475-2875-10-291

**Published:** 2011-10-07

**Authors:** Voravuth Somsak, Chairat Uthaipibull, Parichat Prommana, Somdet Srichairatanakool, Yongyuth Yuthavong, Sumalee Kamchonwongpaisan

**Affiliations:** 1 Protein-Ligand Engineering and Molecular Biology Laboratory, National Center for Genetic Engineering and Biotechnology (BIOTEC), National Science and Technology Development Agency (NSTDA), Thailand Science Park, Pathumthani 12120, Thailand; 2 Department of Biochemistry, Faculty of Medicine, Chiang Mai University, Chiang Mai 50200, Thailand

## Abstract

**Background:**

*Plasmodium vivax *is the most prevalent cause of human malaria in tropical regions outside the African continent. The lack of a routine continuous *in vitro *culture of this parasite makes it difficult to develop specific drugs for this disease. To facilitate the development of anti-*P. vivax *drugs, bacterial and yeast surrogate models expressing the validated *P. vivax *target dihydrofolate reductase-thymidylate synthase (DHFR-TS) have been generated; however, they can only be used as primary screening models because of significant differences in enzyme expression level and *in vivo *drug metabolism between the surrogate models and *P. vivax *parasites.

**Methods:**

*Plasmodium falciparum *and *Plasmodium berghei *parasites were transfected with DNA constructs bearing *P. vivax dhfr-ts *pyrimethamine sensitive (wild-type) and pyrimethamine resistant (mutant) alleles. Double crossover homologous recombination was used to replace the endogenous *dhfr-ts *of *P. falciparum *and *P. berghei *parasites with *P. vivax *homologous genes. The integration of *Pvdhfr-ts *genes via allelic replacement was verified by Southern analysis and the transgenic parasites lines validated as models by standard drug screening assays.

**Results:**

Transgenic *P. falciparum *and *P. berghei *lines stably expressing *Pv*DHFR-TS replacing the endogenous parasite DHFR-TS were obtained. Anti-malarial drug screening assays showed that transgenic parasites expressing wild-type *Pv*DHFR-TS were pyrimethamine-sensitive, whereas transgenic parasites expressing mutant *Pv*DHFR-TS were pyrimethamine-resistant. The growth and sensitivity to other types of anti-malarial drugs in the transgenic parasites were otherwise indistinguishable from the parental parasites.

**Conclusion:**

With the permanent integration of *Pvdhfr-ts *gene in the genome, the transgenic *Plasmodium *lines expressing *Pv*DHFR-TS are genetically stable and will be useful for screening anti-*P. vivax *compounds targeting *Pv*DHFR-TS. A similar approach could be used to generate transgenic models specific for other targets of interest, thus facilitating the development of anti-*P. vivax *drugs in general.

## Background

Current anti-malarial drug development efforts are focused on screening for lead compounds against *Plasmodium falciparum*, the most lethal species. *Plasmodium falciparum *can be routinely cultured *in vitro *and is thus amenable to high throughput compound screening. Anti-malarial drug development against other human *Plasmodium *species, particularly *Plasmodium vivax *- which is the most prevalent cause of human malaria in tropical regions, with an estimated 80 million cases annually [1], is neglected in comparison, since continuous *in vitro *culture methods are not available for these species. The morbidity and mortality caused by *P. vivax *infection are greater than previously believed [2]. Chloroquine has been used as the standard treatment for blood stage *vivax *malaria for more than 40 years; however, chloroquine-resistant *P. vivax *has been reported in many parts of the world [3-5]. Therefore, there is an urgent need for new anti-*vivax *anti-malarial drugs.

One of the validated drug targets for the treatment of malaria infection is dihydrofolate reductase (DHFR; EC1.5.1.3), an essential enzyme for folate biosynthesis [6,7]. The efficacy of current anti-folate drugs targeting *Plasmodium *DHFR is compromised by mutations in the *dhfr *gene, which confer different levels of resistance to these drugs [8-10]. Point mutations in the *P. vivax dhfr *gene equivalent to antifolate resistance mutations found in *P. falciparum *have been associated with antifolate resistance in *P. vivax in vitro *[11-13], leading to the conclusion that wild-type *P. vivax *is sensitive to antifolates, and resistance develops through *dhfr *mutations, similar to the case in *P. falciparum*.

Despite the emergence of drug-resistant *dhfr *mutants, DHFR-TS is still an attractive target for anti-malarial drug development owing to the availability of target-based screening models and crystal structures of both *P. falciparum *[14] and *P. vivax *DHFR enzymes [15]. However, unlike *P. falciparum*, the development of antifolates directed against *P. vivax *has been hampered by the lack of a continuous *in vitro *parasite culture system. Clinical isolates can only be cultured for a short period of time [16,17]. Furthermore, mixed infection with other human malaria parasites can also complicate the drug screening results of clinical isolates. Recently, a *P. vivax in vitro *culture system using erythroblasts has been developed [18], but it is not practical for use in routine drug screening assay.

Surrogate cell expression systems expressing *Pv*DHFR enzymes in yeast [19] and bacteria [20] have been developed as alternatives to parasite drug screening. However, these surrogate systems are of limited use since the level of target enzyme expression and mechanisms of drug metabolism differ markedly from *Plasmodium *parasites. A physiologically similar *Plasmodium *species is clearly a more attractive *P. vivax *surrogate, and this has been proven using transfection technology in which *P. falciparum *parasites expressing mutant *Pv*DHFR-TS have been shown to recapitulate the mutant *Pv*DHFR-TS antifolate resistance [21]. In the same manner, transgenic *P. falciparum *parasites *episomally *expressing drug resistant *Pv*DHFR-TS mutant enzymes have also been developed for studying *P. vivax *drug resistance. However, these transgenic *P. falciparum *parasites still express endogenous *Pf*DHFR-TS as well as episomally expressed *Pv*DHFR-TS enzymes [22], which could confound drug screening results.

In order to facilitate antifolate screening specifically against *Pv*DHFR-TS, transgenic *P. falciparum *and *Plasmodium berghei *parasites stably expressing *Pv*DHFR-TS enzyme replacing the endogenous *Pf *or *Pb*DHFR-TS were generated. These transgenic lines were evaluated as *in vitro *and *in vivo Plasmodium *models for direct assessment of the antifolate efficacy against *Pv*DHFR-TS. These *Plasmodium *surrogate models were thus validated as alternative tools for screening *Pv*DHFR-TS-targeted compounds.

## Methods

### Parasites

*Plasmodium falciparum *strains TM4/8.2 (wild-type DHFR) and K1CB1 (mutant DHFR at residues C59R+S108N) were gifts from Sodsri Thaithong, Faculty of Science, Chulalongkorn University, Thailand. Pyrimethamine-resistant strain K1CB1 was used as parental parasite to generate an *in vitro P. vivax *screening model. The parasites were maintained continuously in human erythrocytes at 37°C under 5% CO_2_, 1% O_2 _and 94% N_2 _in RPMI-1640 culture media supplemented with 24 mM NaHCO_3_, 40 μg/mL gentamicin, 25 mM HEPES and 10% human serum [23]. For generation of *in vivo *screening models, the transgenic *P. berghei *parasite line MRA-867 (PbGFP), a gift from Chris Janse and Andy Waters at Leiden University Medical Center, the Netherlands, was used. This line contains the *gfp *gene stably integrated as a single copy by double cross-over recombination into the *230p *locus, and does not contain a drug selectable marker gene [24,25]. Frozen parasites from stock were mechanically passaged at least once through female BALB/c mice before experiments. Animals were bled from the heart and parasitaemia measured by Giemsa-stained thin blood smear. Infected erythrocytes were suspended in PBS and infection for experiments was carried out by intra-peritoneal injection of approximately 1 × 10^7 ^parasitized erythrocytes.

### Experimental animals

Pathogen-free, six-week-old female BALB/c mice weighing 20-30 g were obtained from the National Laboratory Animal Center, Mahidol University, Thailand. They were kept for at least one week with tap water and pellet diet (CP diet 082, Perfect Companion Company, Bangkok, Thailand) *ad libitum *at 22-25°C and a 12-hour light/12-hour dark cycle. Experiments were started in seven- to eight-week-old animals. Animal experiments were ratified by the Ethical Committee on Animal Experimentation, Faculty of Medicine, Chiang Mai University, Chiang Mai, Thailand and by the Ethical Committee on Animal Experimentation, National Center for Genetic Engineering and Biotechnology (BIOTEC), Thailand. Animal experiments conformed to international and national guidelines for ethical conducts on the care and humane use of animals.

### Construction of transfection plasmids

**Figure 1:**
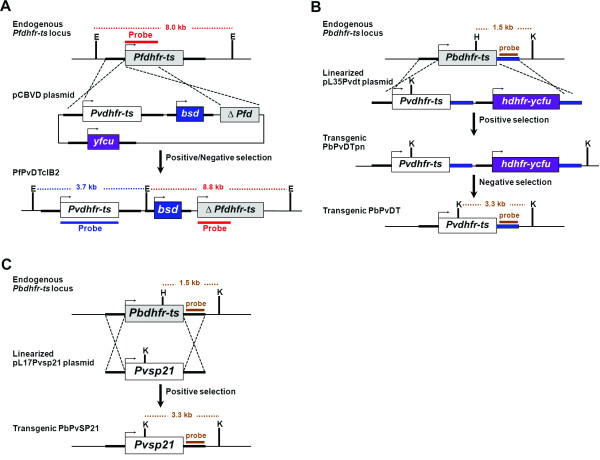
**Diagrammatic representation of strategy to generate transgenic parasites by homologous recombination of *Pvdhfr-ts *gene replacing endogenous *dhfr-ts *locus**. (A) Generation of transgenic *P. falciparum *stably expressing wild-type *Pv*DHFR-TS. The endogenous *Pfdhfr-ts *locus was targeted via double homologous recombination with a plasmid containing expressing cassettes of wild-type *Pvdhfr-ts*, and *blasticidin-S deaminase *(*bsd*) marker. Yeast *cytosine deaminase-uracil phosphoribosyl transferase *(*yfcu*) was used as negative selectable marker. (B) Generation of transgenic *P. berghei *stably expressing wild-type *Pv*DHFR-TS. The endogenous *Pbdhfr-ts *locus was targeted with a linearized plasmid containing expressing cassettes of wild-type *Pvdhfr-ts*, and fusion gene of human *dhfr *(*hdhfr*) and yeast *yfcu *as positive and negative selectable markers respectively. After double homologous recombination, the drug selectable markers are excised by single homologous recombination via the *Pbdhfr-ts *3'UTR repeated sequence elements (blue boxes), whilst retaining the wild-type *Pvdhfr-ts *gene. (C), Generation of transgenic *P. berghei *stably expressing mutant *Pv*DHFR-TS SP21. The endogenous *Pbdhfr-ts *locus was targeted with a linearized plasmid containing expressing cassettes of mutant *Pvdhfr-ts sp21 *(*Pvsp21*) flanked with 5' and 3' UTR of *Pbdhfr-ts *which also serve as the sites for double homologous recombination. Specific probes for Southern analysis are located by bold horizontal line. E: *Eco*RI, H: *Hind*III, K: *Kpn*I.

Wild-type and mutant *P. vivax dhfr-ts *genes were kindly given by Ubolsree Leartsakulpanich, BIOTEC, Thailand [11]. For generation of transgenic *P. falciparum *parasite stably expressing *Pv*DHFR-TS, a transfection plasmid was constructed containing three expression cassettes: 1) *blasticidin-S deaminase *(*bsd*) as a positive selection marker under the control of 5' flanking region of *P. falciparum *camodulin gene (*Pfcam*) and 3' UTR of *P. falciparum *histidine rich protein 2 (*Pfhrp2*) [26], 2) A fusion gene of *cytosine deaminase *and *uracil phosphoribosyl transferase *(*yfcu*) from *Saccharomyces cerevisiae *as a negative selection marker under the control of 5' flanking region of *P. falciparum *heat shock protein 86 gene (*Pfhsp86*) and 3' UTR of *P. berghei *dihydrofolate reductase (*Pbdhfr*) [27], and 3) The wild-type *dhfr-ts *gene of *P. vivax *under the control of 5' flanking region of *Pfdhfr *and 3' UTR of *Pfhrp2 *(Figure 1A). The 5' flanking region and truncated 5' coding sequence of *Pfdhfr-ts *are the sites for homologous recombination and replacement of *Pfdhfr-ts *with *Pvdhfr-ts *gene in the *P. falciparum *parasite chromosome 4.

*Plasmodium berghei *transfection plasmids were constructed from plasmids pL0017 [24] and pL0035 [28], which were kindly provided by Chris Janse and Andy Waters (Leiden University Medical Center, the Netherlands). In order to generate a *P. berghei *parasite line stably expressing wild-type *Pv*DHFR-TS enzyme, transfection plasmids were modified to consist of two expression cassettes (Figure 1B). The first cassette has the wild-type *Pvdhfr-ts *gene flanked by 5' and 3'UTR *Pbdhfr-ts *gene sequences. The second cassette is a drug selection cassette containing a fusion gene of positive (*human dihydrofolate reductase; hdhfr*) and negative (yeast *yfcu*) selectable markers under the control of 5' flanking region of *P. berghei elongation factor 1α-a *(*Pbef1α-a*) and 3'UTR sequence of *Pbdhfr-ts *gene.

For generation of *P. berghei *parasite stably expressing double mutant (S58R+S117N; SP21) *Pv*DHFR-TS enzyme, the transfection plasmid contains the double mutant *Pvdhfr-ts *gene (*Pvsp21*) flanked by the *Pbdhfr-ts *gene 5' and 3' flanking sequences (Figure 1C). The *Pbdhfr-ts *gene sequences serve as the sites for double-crossover homologous recombination. The pyrimethamine-resistant *Pvsp21 *gene itself serves as a positive drug selectable marker for integration and gene replacement.

### Generation of transgenic parasite lines stably expressing *Pv*DHFR-TS

#### *Plasmodium falciparum *transfection

Transfection of *P. falciparum *parasite was performed following a standard protocol [29]. Briefly, synchronized ring stage *P. falciparum *parasite strain K1CB1 (pyrimethamine-resistant) was transfected with 100 µg of transfection plasmid by electroporation (0.2 cm-gap electroporation cuvette, 0.310 kV, 950 µF). The transgenic parasites were first selected with blasticidin. Once blasticidin-resistant parasites were obtained, they were further treated with the negative selectable drug 5-fluorocytosine (5-FC) to select for double crossover integration of *Pvdhfr-ts *gene replacing the endogenous *Pfdhfr-ts *gene. After several rounds of drug cycling, stable transgenic parasites with the desired integration event were obtained and cloned by limiting dilution.

#### *Plasmodium berghei *transfection

Transfection of *P. berghei *parasites was performed using an Amaxa Nucleofector device (Amaxa), following the standard protocol [30]. Purified schizonts of GFP-expressing *P. berghei *ANKA parasite line MRA-867 (PbGFP) were transfected with 10 µg of linearized transfection plasmid in 100 µl of Human T Cell Nucleofector solution (Amaxa) using programme U-33, and then injected intravenously into naïve BALB/c recipient mice. Twenty-four hours post-injection, blood smears were made from the host animals and the presence of parasites checked by Giemsa staining. Drug-resistant parasites were selected by pyrimethamine administered to the drinking water (70 µg/ml). Under this selection regimen, *Pv*DHFR-TS SP21 conferred pyrimethamine resistance to transgenic parasites. For selection of *P. berghei *harbouring wild-type *Pvdhfr-ts*, after pyrimethamine selection for the positive drug marker *hdhfr*, infected mice were injected intra-peritoneally twice a day with 5-FC (dissolved in 0.9% NaCl) at 1 g/kg bodyweight for a period of three days to select for parasites in which the drug selectable markers had been excised by single homologous recombination via the *Pbdhfr-ts *3'UTR repeated sequence elements [28], whilst retaining the wild-type *Pvdhfr-ts *gene. The *Pv*DHFR-TS-expressing transgenic *P. berghei *parasites were subsequently cloned by the method of limiting dilution [31].

### Characterization of transgenic parasites

Correct integration of the constructs into the host's endogenous *dhfr-ts *locus was demonstrated by Southern analysis of restricted genomic DNA from transgenic parasites. Genomic DNA from erythrocytes infected with transgenic *P. falciparum *harbouring *Pvdhfr-ts *gene was purified using a QIAamp Blood Mini Kit (Qiagen) according to the manufacturer's instructions. Blood from mice infected with transgenic *P. berghei *harbouring *Pvdhfr-ts *or *Pvsp21 *genes was collected by cardiac puncture, and then depleted of leucocytes by passing through a CF11 cellulose column (Whatman). Following lysis of the erythrocyte pellet with 0.2% (w/v) saponin in PBS, total genomic DNA from parasites was obtained using a QIAamp Blood Mini Kit (Qiagen). The resulting DNA was then used for Southern analysis to confirm allelic replacement of the endogenous *dhfr-ts *with the *P. vivax *homologue. Briefly, 30 µg of genomic DNA from transgenic parasites was restriction-digested, separated in 0.6% (w/v) agarose gel and transferred onto a Hybond N+ nylon membrane (GE Healthcare) by capillary blotting. Digested DNA was fixed to the membrane by UV cross-linking and hybridized with specific probes prepared with a DIG labelling kit (Roche Applied Science). All probes used in this study are shown schematically in Figure 1. The pattern of hybridization was detected using alkaline phosphatase-conjugated anti-DIG antibody and CSPD reagent according the manufacturer's instructions (Roche Applied Science).

### Validation of transgenic parasites as models for *Pv*DHFR-TS screening

#### SYBR Green I assay

*Plasmodium falciparum *strains TM4/8.2, K1CB1 and transgenic *P. falciparum *expressing *Pv*DHFR-TS were maintained in human erythrocytes as previously described and synchronized to 1% ring stages at 2% haematocrit. Ninety microliters of cultured parasites were transferred to each well of a 96-well microtitre plate before 10 μl of pyrimethamine (Sigma), chloroquine (Sigma) and dihydroartemisinin (a gift from Dafra Pharma International) at different concentrations were added in triplicate. The mixtures were incubated at 37°C under 5% CO_2_, 1% O_2 _and 94% N_2 _for 48 hours. To determine parasite growth, 100 μl of SYBR green I (at 1:5,000 dilution in culture media; Invitrogen) was added to the culture mixtures for 30 minutes at room temperature in the dark [32]. The SYBR green I-stained parasitized erythrocytes were then analysed using a Cytomics 500 FC MPL flow cytometer (Beckman Coulter) equipped with a 96-well plate adaptor. Erythrocytes were identified on the basis of their specific forward (FSC) and side (SSC) light-scattering properties. The green fluorescence signal was excited with an argon ion laser at a wavelength of 488 nm and the emission of the green fluorescence was detected using a 530/30 nm band pass filter. A previous analysis of non-infected erythrocytes was performed to determine a cut off for these cells to distinguish them from parasitized cells. The fluorescent intensity of a total of 100,000 events per sample was measured and data analysis was subsequently performed using CXP software (Beckman Coulter). For calculation of the percentage of parasite growth, the mean parasitaemia of untreated parasites was set at 100% growth. The concentration of drugs that inhibits 50% of the parasite growth (IC_50_) was determined from the sigmoidal curve obtained by plotting the percentages of parasite growth against drug concentrations.

#### GFP assay

In order to validate transgenic *P. berghei *parasite stably expressing *Pv*DHFR-TS enzymes as *in vivo *models for antifolate drug screening, the assessment of anti-malarial drug efficacy *in vivo *was performed using the standard four-day suppressive test [33]. Groups of at least three mice were inoculated with approximately 1 × 10^7 ^parasitized erythrocytes intravenously and then treated with standard anti-malarials: pyrimethamine (Sigma), chloroquine (Sigma) or artesunate (Mekophar Chemical Pharmaceutical) once daily for four successive days from the day of parasite inoculation. The drugs at chosen doses (mg/kg of body weight) were freshly prepared in 100% DMSO (pyrimethamine and artesunate) or distilled water (chloroquine) and administered orally by gavage. It was found that pyrimethamine at 50 mg/kg was lethal to mice as all animals died soon after treatment with this dose by oral administration (data not shown). Hence, the maximum pyrimethamine dose was 30 mg/kg. Untreated controls were given either distilled water or DMSO only. The course of parasitaemia in untreated mice (control group) and in mice treated with different doses of drugs was monitored by flow cytometry since these parasites express GFP as fluorescence marker. A drop of blood was collected directly into wells of a 96-well microtitre plate containing 200 µl of RPMI-1640 culture media and heparin (1U/µl). The GFP signal in these samples was measured as described above. For calculation of the growth inhibitory curves, the mean parasitaemia of the control samples was set at 0% inhibition. The concentration of drugs that inhibits 50% of the parasite growth (ED_50_) was determined from the sigmoidal curve obtained by plotting the percent parasite growth inhibition against drug concentrations.

### Statistical analysis

Parasites' growth and growth inhibitory curves and statistical analysis of the data were performed using the GraphPad Prism software version 5.0 (GraphPad Software). The non-linear regression function for sigmoidal dose-response (variable slope) was used to calculate the best-fit 50% inhibition concentration (IC_50_) or 50% effective concentration (EC_50_) values.

## Results

### Generation of transgenic *Plasmodium falciparum *harbouring *Pvdhfr-ts *gene

**Figure 2:**
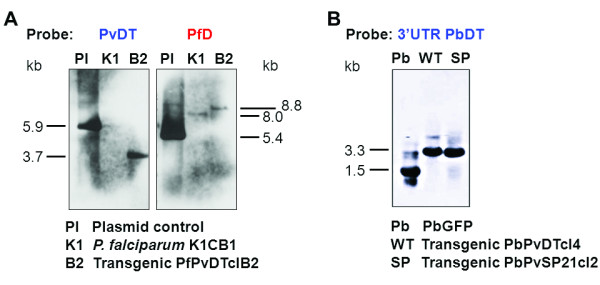
**Southern analysis of transgenic parasites to confirm allelic replacement of *dhfr-ts *of *Plasmodium falciparum *(A) and *Plasmodium berghei *(B) with *dhfr-ts *gene from *P. vivax***. DNA probes specific to *Pvdhfr-ts *(PvDT), *Pfdhfr *(PfD) and 3'UTR of *Pbdhfr-ts *(3'UTR PbDT) were used to detect restriction-digested fragments from genomic DNA of transgenic parasites. Pl: pCBVD plasmid control; K1: *P. falciparum *K1CB1; B2: Transgenic PfPvDTclB2; PbGFP: *P. berghei *GFP; WT: transgenic PbPvDTcl4; SP: transgenic PbPvSP21cl2.

Pyrimethamine-resistant *P. falciparum *strain K1CB1 was transfected with plasmid DNA for replacement of the endogenous *Pfdhfr-ts *with wild-type *Pvdhfr-ts*. Following rounds of positive and negative selections, transgenic *P. falciparum *were obtained. A clonal parasite line designated PfPvDTclB2 was obtained and gene replacement verified by Southern blotting analysis. As shown in Figure 2A, hybridization with a *Pvdhfr-ts *DNA probe showed the expected 3.7 kb and 5.9 kb *Eco*RI-digested fragments from transgenic PfPvDTclB2 genomic DNA and control plasmid DNA, respectively, confirming the integration of *Pvdhfr-ts *at the correct site, while no signal was detected for parental *P. falciparum *K1CB1 line. Another DNA probe specific to *Pfdhfr *could detect the expected 8.8 kb, 8.0 kb and 5.4 kb *Eco*RI-digested fragments from genomic DNA of transgenic PfPvDTclB2, parental *P. falciparum *K1CB1 line and control plasmid DNA, respectively. From these results, it can be concluded that transgenic PfPvDTclB2 parasites stably express wild-type *Pvdhfr-ts *instead of endogenous *Pfdhfr-ts*; furthermore, these parasites are clonal, lack episomal plasmid DNA and can be stably maintained without drug selection.

### Generation of transgenic *Plasmodium berghei *harbouring *Pvdhfr-ts *gene

Blood stages of the reference GFP-expressing *P. berghei *parasite line PbGFP were transfected with the linear form of the transfection vectors by electroporation in order to introduce either wild-type *Pvdhfr-ts *or mutant *Pvsp21 *genes replacing the endogenous *Pbdhfr-ts *gene. For selection of *P. berghei *harbouring wild-type *Pvdhfr-ts*, a two-step drug selection procedure was performed. First, transgenic parasites with integrated wild-type *Pvdhfr-ts *were selected with pyrimethamine. Subsequently, negative selection was performed to select for parasites which had excised the positive-negative drug selection cassette in order to obtain parasites that have only wild-type *Pvdhfr-ts *gene integrated to the *P. berghei *genome (Figure 1B). Transgenic *P. berghei *parasite clones stably expressing wild-type *Pv*DHFR-TS (designated PbPvDTcl4) or double mutant *Pv*DHFR-TS SP21 (designated PbPvSP21cl2) were obtained and analysed by Southern blotting. As shown in Figure 2B, the *Kpn*I/*Hind*III digested genomic DNA fragments corresponding to the parental wild type and transgenic *P. berghei *parasites (1.5 kb and 3.3 kb, respectively) were detected by a DNA probe specific to 3'UTR *Pbdhfr-ts*. Thus, this confirmed the successful generation of transgenic *P. berghei *parasites stably harbouring wild-type *Pvdhfr-ts *or mutant *Pvsp21*, replacing the endogenous *Pbdhfr-ts *gene.

### Drug sensitivity analysis of transgenic *Plasmodium falciparum *stably expressing wild-type *Pv*DHFR-TS enzyme

**Figure 3:**
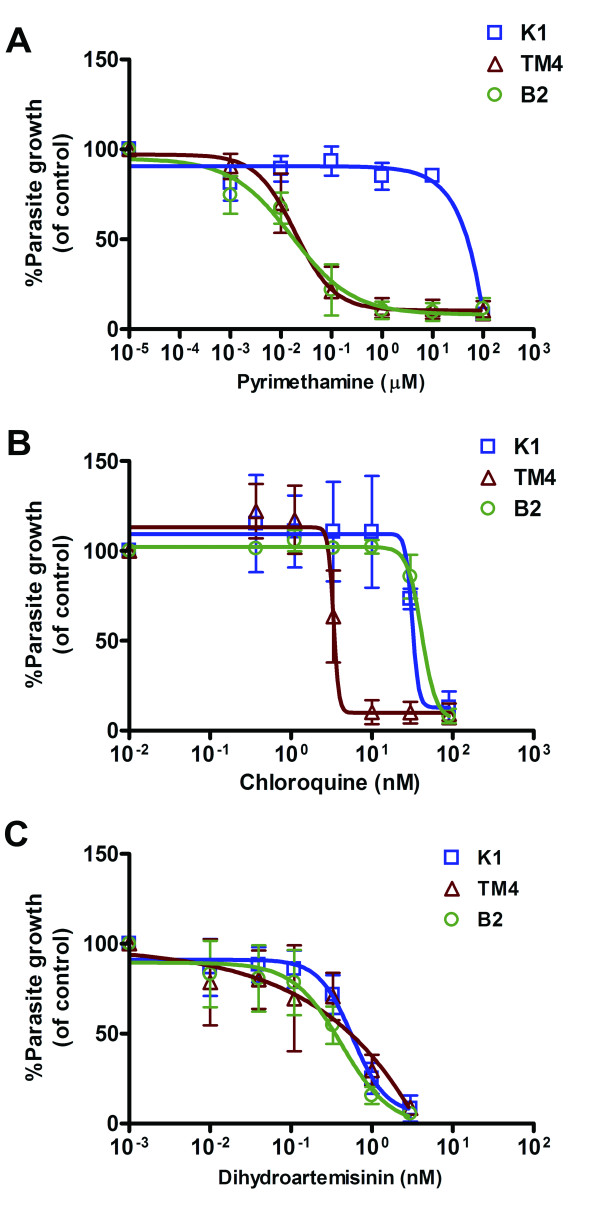
**Sensitivity of transgenic *Plasmodium falciparum *expressing wild-type *Pv*DHFR-TS enzyme to pyrimethamine (A), chloroquine (B) and dihydroartemisinin (C)**. The growth of parasites treated with pyrimethamine, chloroquine and dihydroartemisinin was detected using the SYBR Green I staining assay. The percentage of parasite growth was plotted against drug concentrations. Data were shown as mean ± S.D. of at least 3 independent experiments. K1: *P. falciparum *K1CB1; TM4: *P. falciparum *TM4/8.2; B2: transgenic PfPvDTclB2.

Transgenic PfPvDTclB2 parasite was evaluated against standard anti-malarial drugs. Pyrimethamine is the standard antifolate drug and used as the primary compound to validate this system. As shown in Figure 3A and Table 1, the transgenic PfPvDTclB2 parasite was much more sensitive to pyrimethamine than the parental K1CB1 line, verifying that the wild-type *Pvdhfr-ts *gene replacing the *Pfdhfr-ts *gene is a pyrimethamine-sensitive variant. Moreover, the level of pyrimethamine sensitivity in the transgenic PfPvDTclB2 parasite is the same as the antifolate-sensitive *P. falciparum *TM4/8.2 strain (IC_50 _= 0.03 ± 0.02 µM). The parental K1CB1 line is also resistant to chloroquine, a 4-aminoquinoline drug that inhibits haemozoin formation in the food vacuole of the parasites. This transgenic PfPvDTclB2 line shows the same chloroquine-resistant phenotype as the parental *P. falciparum *K1CB1 strain, with IC_50 _values of 49.5 ± 5.8 nM and 46.0 ± 3.1 nM respectively (Figure 3B and Table 1), indicating that the *dhfr-ts *gene replacement did not affect sensitivity to drugs not targeting DHFR-TS. Another non-antifolate drug control used in this study was dihydroartemisinin (DHA). All parasites tested in this study were sensitive to DHA at the IC_50 _values of 0.6 ± 0.1 nM, 0.7 ± 0.3 nM and 0.4 ± 0.1 nM for *P. falciparum *TM4/8.2, K1CB1 and transgenic PfPvDTclB2, respectively (Figure 3C and Table 1).

**Table 1:** Drug sensitivity of transgenic *Plasmodium *expressing *Pv*DHFR-TS to standard anti-malarials

*P. falciparum*		IC_50 _± S.D.	
	
	Pyrimethamine (µM)	Chloroquine (nM)	Dihydroartemisinin (nM)
*P. falciparum *TM4/8.2	0.03 ± 0.02	4.3 ± 1.4	0.6 ± 0.1
*P. falciparum *K1CB1	28.7 ± 1.7	46.0 ± 3.1	0.7 ± 0.3
PfPvDTclB2	0.03 ± 0.02	49.5 ± 5.8	0.4 ± 0.1

***P. berghei***		**ED_50 _± S.D. (mg/kg)**	
	
	**Pyrimethamine**	**Chloroquine**	**Artesunate**

*P. berghei *GFP	0.69 ± 0.21	1.56 ± 0.12	5.43 ± 0.42
PbPvDTcl4	0.53 ± 0.24	2.85 ± 0.17	7.43 ± 0.30
PbPvSP21cl2	> 30	3.88 ± 0.13	7.59 ± 0.33

### Drug sensitivity analysis of transgenic *Plasmodium berghei *stably expressing *Pv*DHFR-TS enzymes

**Figure 4:**
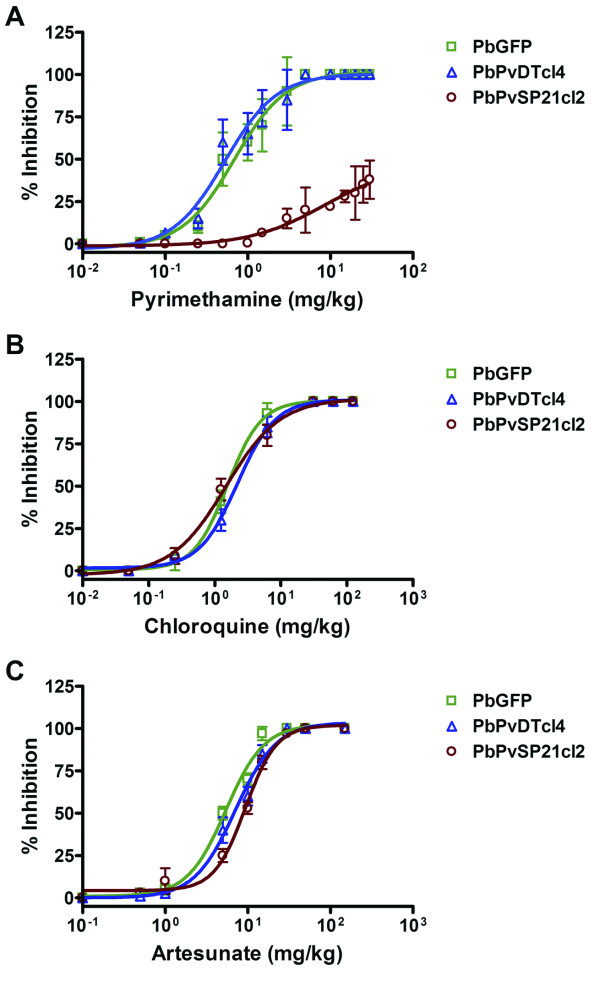
**Sensitivity of transgenic *Plasmodium berghei *expressing *Pv*DHFR-TS enzyme to pyrimethamine (A), chloroquine (B) and artesunate (C)**. The transgenic parasites were validated using the standard four-day suppressive test. Percent parasite inhibition was plotted for groups of at least five mice orally given varying doses of pyrimethamine, chloroquine and artesunate. Data were shown as mean ± S.D. of at least three independent experiments.

After inoculation, mice in the untreated control group showed a progressively increasing parasitaemia, and all the mice died by day 11 (data not shown). As shown in Figure 4A and Table 1, the transgenic PbPvDTcl4 demonstrated a drug susceptibility profile similar to that of the wild-type parental PbGFP with an ED_50 _of 0.53 ± 0.24 mg/kg and 0.69 ± 0.21 mg/kg, respectively. This demonstrated that the wild-type *Pv*DHFR-TS enzyme was equally susceptible to the antifolate compound compared with wild-type *Pb*DHFR-TS. In contrast, transgenic PbPvSP21cl2 was approximately 40-fold more resistant to pyrimethamine than the PbPvDTcl4 parasite line (Figure 4A). Therefore, the double mutant *P. vivax *DHFR-TS confers a high level of resistance to pyrimethamine in *P. berghei.*

The transgenic *P. berghei *lines were also tested with the non-antifolate drugs chloroquine and artesunate. As shown in Figure 4B and Table 1, all parasite lines were similarly susceptible to chloroquine. The ED_50 _values against chloroquine were 1.56 ± 0.12 mg/kg, 2.85 ± 0.17 mg/kg, and 3.88 ± 0.13 mg/kg mg/kg in parental PbGFP, PbPvDTcl4 and PbPvSP21cl2 parasites, respectively. Artesunate is extremely potent against pyrimethamine-resistant parasites. It is a semi-synthetic derivative of artemisinin that is water-soluble and may therefore be given by injection. All parasite lines were also susceptible to artesunate treatment with ED_50 _values of 5.43 ± 0.42 mg/kg, 7.43 ± 0.30 mg/kg and 7.59 ± 0.33 mg/kg in parental PbGFP, PbPvDTcl4 and PbPvSP21cl2 parasites, respectively (Figure and Table 1).

## Discussion and Conclusions

This study describes the generation of both *in vitro *and *in vivo *transgenic *Plasmodium *parasites stably expressing *P. vivax *DHFR-TS enzyme and the application of these parasites for assessing antifolate compound efficacy. The transgenic *Plasmodium *parasites harbour only one copy of *Pvdhfr-ts *gene, which is under the control of the endogenous *dhfr-ts *regulatory sequences. The fact that it is possible to replace the endogenous *Plasmodium dhfr-ts *with a homologue from a different species suggests that *dhfr-ts *function is conserved among species of this genus. This is in agreement with biochemical evidence that cross-species heterodimers of *Plasmodium *DHFR-TS are fully functional [34]. While the expression of double mutant *Pv*DHFR-TS SP21 was not deleterious and can support the growth of the transgenic *P. berghei *parasites, attempts to obtain *P. falciparum *stably expressing double mutant *Pv*DHFR-TS SP21 were not successful (data not shown). The reasons may be the catalytic activity of *Pv*DHFR-TS SP21 is insufficient to support the growth of transgenic *P. falciparum*. It was reported that the catalytic activity, as determined by *k*_cat_/*K*_m of H2folate_, of *Pv*DHFR SP21 is approximately 13 times and 27 times lower than that of wild-type *Pv*DHFR [11] and wild-type *Pf*DHFR [35], respectively.

A major advantage of the *Pvdhfr-ts *transgenic parasites generated in the study is that the gene is stably maintained in an integrated fashion. This issue is important since it has been reported that the apparent copy number of plasmid DNA in episomally transfected *Plasmodium *is variable, which may affect the drug sensitivity profile in drug testing assays. The reasons for varying copy numbers are related to the nature of the episomal plasmid itself and the level and duration of drug pressure [36]; moreover, higher drug concentration are known to select for increased copy numbers of the episome [37]. In the absence of drug pressure, both transgenic *P. falciparum *and *P. berghei *lose episomal transgenes [36,38,39]; hence, constant drug pressure is needed to maintain the episomes. In the earlier report of *P. falciparum *episomally expressing *Pvdhfr-ts*, no effect of episomal copy number on the drug sensitivity profile was observed, probably owing to the tight regulation of the parasite DHFR-TS expression level [22,40]. However, the episomal copy number did vary since the drug used to maintain the episome must be withdrawn for compound testing, hence the episomal system may not be sufficiently robust as a general drug screening method.

In this study, *in vitro *and *in vivo Plasmodium *models have been generated specifically for anti-*Pv*DHFR screening. There are reports that compounds that have anti-*P. falciparum *activity in *in vitro *screening do not show the same anti-*Plasmodial *activity in *in vivo *models, such as *P. berghei *[41,42]. It is therefore premature to assume that compounds found to be active *in vitro *will also be efficacious *in vivo *before pharmacokinetic studies of the compounds in animal models have been conducted, since compounds with poor pharmacokinetic properties may simply not reach their targets. At present, the only *in vivo *model for *P. vivax *is *P. cynomolgi *infecting primates [43]. This model is expensive and restricted to specialized laboratories; hence the transgenic *P. berghei *expressing *P. vivax *targets is an attractive alternative experimental model.

In conclusion, transgenic *P. falciparum *and *P. berghei *stably expressing *Pv*DHFR-TS replacing the endogenous *Plasmodium dhfr-ts *were successfully generated as *in vitro *and *in vivo Plasmodium *drug screening models. The drug sensitivities of the transgenic lines varied according to the introduced *Pvdhfr-ts *gene. Until a routine continuous *in vitro *culture system is developed for *P. vivax*, these transgenic models will be useful for screening of anti-*P. vivax *compounds targeting the *Pv*DHFR enzyme. A similar approach could be used to generate transgenic models specific for other targets of interest, thus facilitating the development of anti-*P. vivax *drugs in general.

## Competing interests

The authors declare that they have no competing interests.

## Authors' contributions

VS, CU, SS, YY and SK conceived and designed the project. VS, CU and PP performed experiments. VS and CU prepared the manuscript. SS, SK and YY critically reviewed the manuscript. All authors read and approved the final manuscript.
